# Amiodarone induces cell wall channel formation in yeast *Hansenula polymorpha*

**DOI:** 10.1186/s40064-015-1185-2

**Published:** 2015-08-26

**Authors:** Tatyana S. Kalebina, Sviatoslav S. Sokolov, Irina O. Selyakh, Darya P. Vanichkina, Fedor F. Severin

**Affiliations:** Department of Molecular Biology, Faculty of Biology, Lomonosov Moscow State University, 1-12 Leninskie Gory, Moscow, 119992 Russia; Belozersky Research Institute of Physico-Chemical Biology, Lomonosov Moscow State University, 1-40 Leninskie Gory, Moscow, 119992 Russia; Department of Bioengineering, Faculty of Biology, Lomonosov Moscow State University, 1-12 Leninskie Gory, Moscow, 119992 Russia; Institute for Molecular Bioscience, University of Queensland, Queensland, Australia

**Keywords:** Programmed cell death, Yeast cell wall, Amiodarone

## Abstract

The yeast cell wall is constantly remodeled to enable cell growth and division. In this study, we describe a novel type of cell wall modification. We report that the drug amiodarone induces rapid channel formation within the cell wall of the yeast *Hansenula polymorpha*. Light microscopy shows that shortly after adding amiodarone, spherical structures, which can be stained with DNA binding dyes, form on the cell surface. Electron microphotographs show that amiodarone induces the formation of channels 50–80 nm in diameter in the cell wall that appear to be filled with intracellular material. Using fluorescent microscopy, we demonstrate MitoTracker-positive DNA-containing structures visibly extruded from the cells through these channels. We speculate that the observed channel formation acts to enable the secretion of mitochondrial material from the cell under stressful conditions, thus enabling adaptive changes to the extracellular environment.

## Background

The yeast cell wall (CW) is a dynamic, multifunctional, and physiologically active organelle that is responsible for interactions between the microorganism and its environment (Lesage and Bussey 2006; Orlean 2012; Teparić and Mrsa 2013).

The major components of the CW are glucan, mannoproteins, and chitin. Glucan consists of β-1,3- and β-1,6-bound glucose residues and is the central structural component (Klis et al. 2002) that forms a rigid network to which other functional molecules, particularly mannoproteins, are covalently bonded. During yeast cell growth and division, the CW is constantly remodeled (Sestak et al. 2004). The remodeling process is carried out by glucan remodeling enzymes and includes hydrolysis of the polysaccharides network and subsequent incorporation of newly synthesized molecules.

Programmed cell death has been extensively studied in yeasts. In particular, it has been shown that the drug amiodarone can induce cell death in *Saccharomyces cerevisiae* by triggering a cascade of proapoptotic changes in mitochondria. Amiodarone stimulates respiration and increases energy coupling in mitochondria, and this leads to generation of reactive oxygen species (Pozniakovsky et al. 2005; Sokolov et al. 2006). It is well known that the rigid cell wall is essential for the protection of yeast cells against negative environmental factors. Earlier, it was demonstrated that amiodarone affects a number of genes involved in CW synthesis (Courchesne et al. 2009; Gamarra et al. 2010). Therefore, one can expect that amiodarone would cause certain structural changes in the CW. However, there are no data on morphological changes in the structure of the cell wall of yeast that occur under the influence of amiodarone.

Over the years, we have accumulated significant experience in treatment of *S. cerevisiae* cells with various toxic, especially pro-oxidant agents, and we have never seen membranous blebs being extruded by the cell—the phenomenon described in this work. Interestingly, similar changes were reported for diamide-treated *S. cerevisiae* cells (de Souza Pereira and Geibel 1999). Using atomic-force microscopy, they revealed structures in the cell wall that they called “pores”. Unfortunately, the data provided by their report seem to be not sufficiently conclusive due to limitations of their technique. Because atomic-force microscopy does not allow determining whether the pores span the cell wall, we think that most likely in this case there were thin layers of cell wall material at the bottom of the pores.

Thus, we studied the effect of amiodarone on a similar model organism, the budding obligate aerobe yeast *Hansenula polymorpha*^1^. We found that high doses of amiodarone (lethal for approximately half of the cells present in the culture) induced dramatic morphological changes in the *H*. *polymorpha* cell wall leading to formation of channels. After amiodarone treatment, the MitoTracker-positive cellular content of *H. polymorpha*, presumably mitochondria, extruded through these channels.

## Results and discussion

**Fig. 1:**
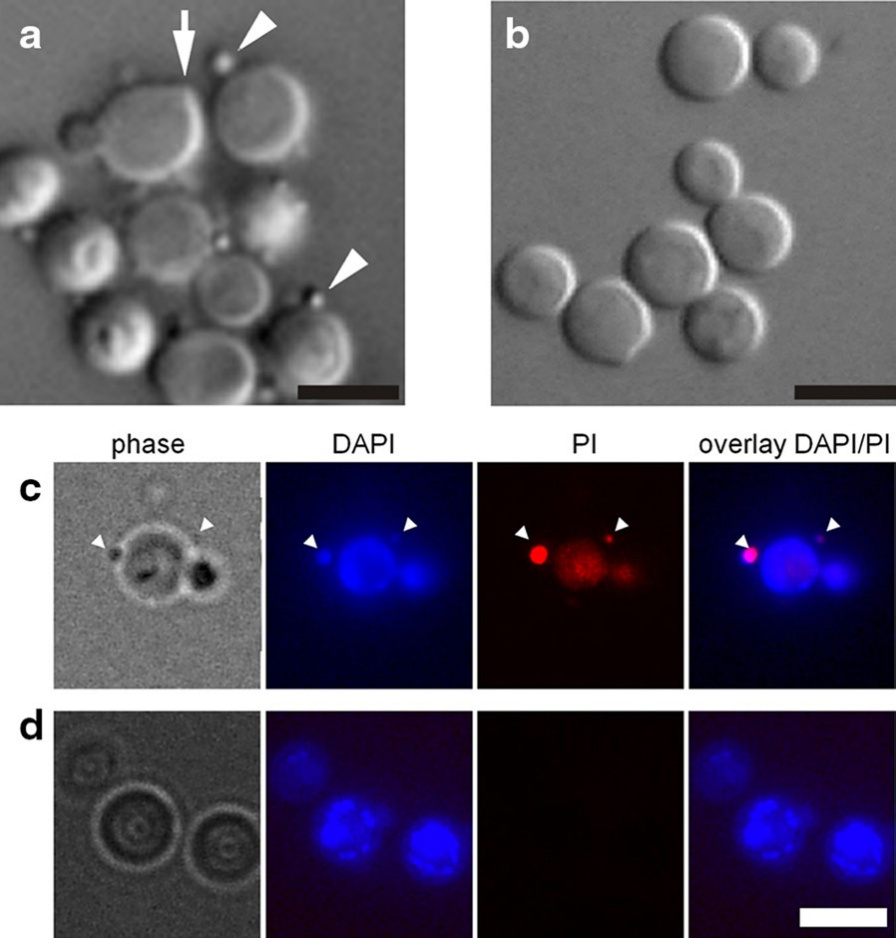
*Hansenula polymorpha* cells treated with amiodarone (**a**, **c**) or untreated cells (**b**, **d**). **a**, **b**—differential interference contrast (DIC) microscopy, **c**, **d**—combined phase and fluorescence microscopy images of cells after propidium iodide and DAPI treatment. The *arrow* marks a cone-shaped protuberance (**a**); *arrowheads* mark spherical structures at the surface (**a**, **c**). *Bar length* 5 μm

**Fig. 2:**
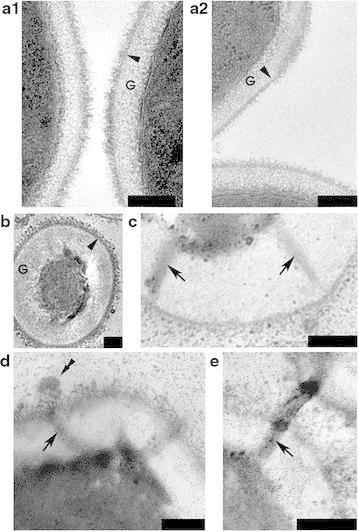
Electron microphotographs of untreated *Hansenula polymorpha* cells (**a**) and cells treated with amiodarone (**b**–**e**). Electron-dense channels (*black arrow*) span the cell wall. *G* glucan layer, *black arrowheads*—mannoproteins. Electron-dense areas can be seen where the channels exit the cell (*double black arrowheads*). *Bar length* 0.2 μm

To see the effect of amiodarone on *H. polymorpha*, we treated cells grown in a liquid culture with varying concentrations of the drug and found that amiodarone at 80 μM concentration killed approximately 50 % of the cells. Using differential interference contrast (DIC) microscopy, we noticed that after addition of 80 μM amiodarone, cells formed cone-shaped protrusions and spherical structures at their surfaces (Fig. 1a, b) after 15 min of incubation with amiodarone. To determine the nature of these structures, we stained them with a number of fluorescent dyes. We found that both propidium iodide (PI) and 6′-diamidino-2-phenylindole (DAPI) (Fig. 1c, d) were accumulated by these spherical structures. Our observations suggested the intriguing possibility that DNA was being transported across the cell wall. We reasoned that dramatic changes must occur in cell wall morphology, and we used transmission electron microscopy (EM) to test this hypothesis. Electron microphotographs revealed that amiodarone triggered a number of structural changes in the cell wall: (1) the wall visibly thickened in an uneven way: (2) the electron-transparent glucan layer (marked G, Fig. 2a, b) thickened up to 200–400 nm, 2–4 times thicker than in the control; (3) the electron-dense external layer, supposedly of mannoprotein nature (Fig. 2b), becomes less dense and seemed thinner compared to a glucan layer (Fig. 2a–c). Crucially, during this process the cell did not change its size significantly (Fig. 1a, b). We also noticed that unusual structures were forming within the cell walls, which we called channels, because they spanned the cell wall (Fig. 2c–e). We estimate the diameter of the channels to be 50–80 nm. Interestingly, intracellular material appeared to be transferred across these channels (Fig. 2c–e).

The electron microscopy study showed that the intracellular material gathered in the proximity of the channels (Fig. 2d) and formed electron-dense regions (Fig. 2d). The images suggest that after passing through the channel, this material accumulated at the cell wall outer surface (Fig. 2d) forming spherical structures. Moreover, the EM images suggest that the channels of neighboring cells might interact with each other to allow the exchange of the intracellular material (Fig. 2e). Such contacts are most likely destroyed in a liquid medium because the flow of the liquid ruptures the contacts.

Our results raise two questions. First, what is the physiological reason for formation of the channels? The fact that the material protruding from them was positively stained by PI and DAPI points to the presence of DNA in this material, suggesting that amiodarone induced extrusion of DNA from the cell through the channels. The origin of those DNA molecules could be either nuclei or mitochondria.

It was suggested by Koren (2006) that stressful conditions might induce gene transfer between yeast cells. He argued that mixing of the genetic material might be beneficial to the cell population under severe stress (Koren 2006).

We monitored the transfer of two different auxotrophy markers between cells upon amiodarone treatment. We estimated the frequencies of gene transfer by using colony growth assay on selective media lacking both auxotrophic substances. Equal amounts of m14 (*leu2ade2URA3*) and u23m25 (*leu2ura3ADE2*) cells were mixed, and amiodarone was added to the mixture. After 5 min, the cells were centrifuged to facilitate cell-to-cell contacts and then incubated for 1 h. The cells were then resuspended and transferred onto YNB plates lacking adenine and uracil. The control cells were treated in the same way but excluding addition of amiodarone. In approximately 50 experiments, it was seen that the control cells did not produce any colonies, while the amiodarone-treated mixtures of the two cell types produced colonies with an estimated frequency of approximately 10^−9^ viable colonies per total number of cells in the assay. This estimate came from the following data: we observed 11 colonies in approximately 50 experiments, and the number of cells in the experiments was in the range of 10^7^–10^8^.

**Fig. 3:**
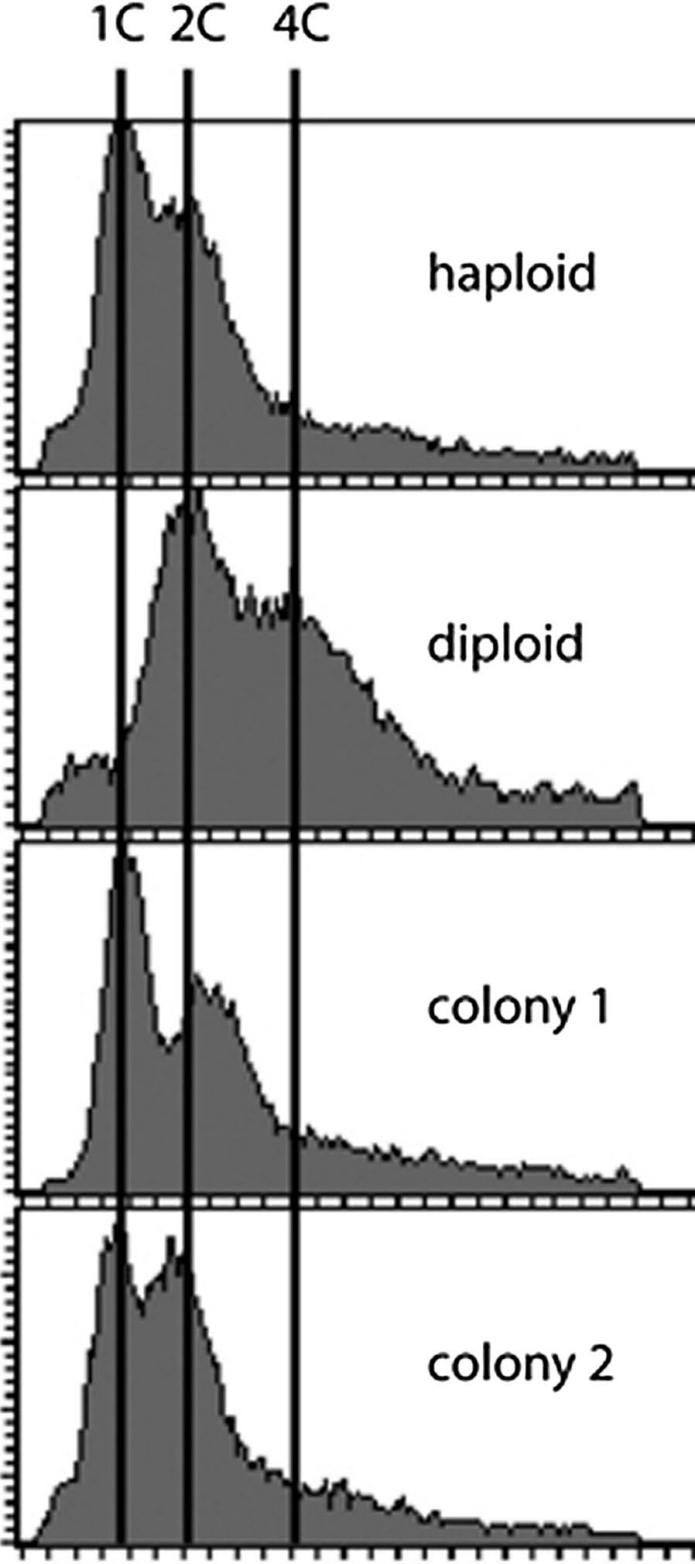
FACS profiles of DNA content of *Hansenula polymorpha* haploid and diploid strains and two colonies obtained after amiodarone treatment grown on media without adenine and uracil. Axis X: cellular DNA content. Axis Y: Relative frequency. C1, C2, and C4—DNA content

To confirm that the gene transfer was not due to conventional mating, we checked using a FACS assay whether the surviving colonies contained haploid or diploid cells. The cells were haploid, which excludes the possibility that the marker acquisition was due to mating. Seven colonies were analyzed, and all of them appeared to be haploid; two representative FACS profiles are shown (Fig. 3). Therefore, our experimental results are consistent with the idea of transfer of genetic markers between the cells, but the low frequency of the transformation leaves the possibility that the ability to grow was acquired via random mutagenesis.

**Fig. 4:**
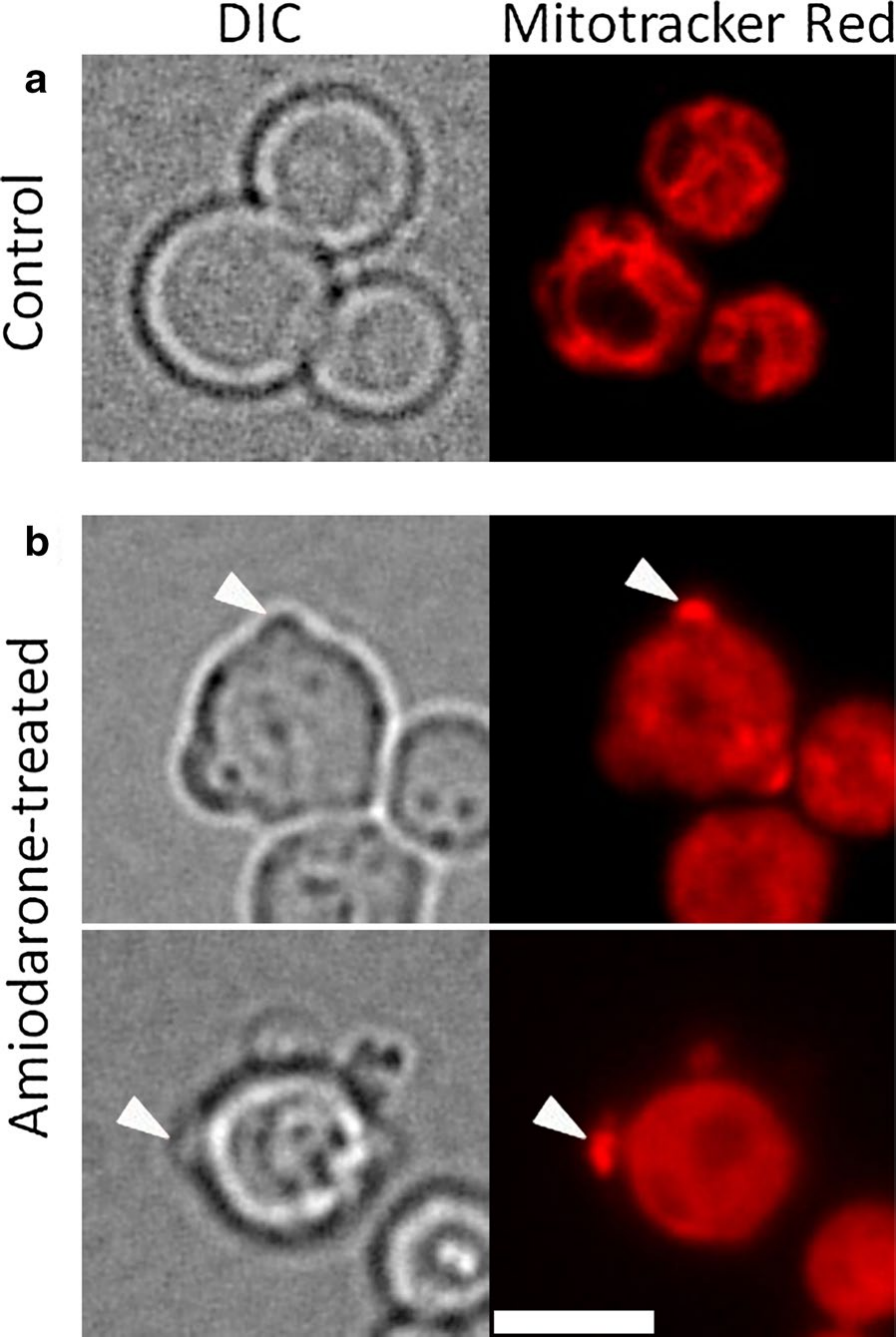
Untreated *Hansenula polymorpha* cells (**a**) or cells treated with amiodarone (**b**). *Left*—DIC microscopy; *right*—fluorescence microscopy images of cells after Mitotracker Red treatment. *Arrowheads* show a Mitotracker-positive protrusion. All pictures are of the same scale. *Bar length* 5 μm

At the same time, it is known that the formation of mitoptotic bodies and extrusion of mitochondrial material from the cell (mitoptosis) serves as a protection of eucaryotic cells under conditions of mitochondrial stress (Lyamzaev et al. 2008). Thus, it is tempting to suggest that, similar to stressed mammalian cells, in yeasts amiodarone stimulates mitoptosis, and the cells get rid of mitochondria through the channels in the cell wall. We observed MitoTracker-positive structures that were visibly detected as protrusions on the cell surface after amiodarone treatment (Fig. 4). It also can be seen that the MitoTracker staining is more diffuse in the treated cells. This is not surprising: in *S. cerevisiae*, high concentrations of amiodarone cause major changes in mitochondria: at first, they hyperpolarize them, inducing fragmentation, and later mitochondria depolarize and rupture (Pozniakovsky et al. 2005; Ozhovan et al. 2009). We speculate that the PI- and MitoTracker-stained extracellular material identified as mitochondrion-like particles surrounded by a membrane were indeed mitochondria, and the observed process was mitoptosis.

The second question concerns the mechanism of such a rapid channel formation in the *H. polymorpha* cell wall. It is possible that enzymes with autolytic substrate specificity digest the cell wall to produce the channels. Indeed, yeast cells can make considerable adjustments to the composition and structure of the wall, for example during the cell cycle or in response to environmental conditions such as nutrient and oxygen availability (Smits et al. 1999). These changes are caused by periplasmic or cell wall enzymes, most notably the glucan remodeling enzymes that remodel the β-glucans—the main constituents of the cell wall (Smits et al. 1999; Cabib et al. 2008). Our data are consistent with results obtained previously (Courchesne et al. 2009). The formation of “canals” was demonstrated after cultivation of the yeast *Schwanniomyces occidentalis* on hydrocarbons (Dmitriev et al. 1980) and was shown to be accompanied by activation of β-glucosidase, β-glucanase, and α-mannosidase—enzymes capable of degrading cell wall polysaccharides (Dmitriev et al. 1980). Further research is required to determine whether the amiodarone-induced channels that we observed formed due to rapid activation of these autolytic enzymes.

## Conclusions

To summarize, our data and previous observations (Dmitriev et al. 1980; Pozniakovsky et al. 2005; Sokolov et al. 2006; Lyamzaev et al. 2008; Courchesne et al. 2009) are consistent with the idea that the presence of amiodarone in the medium creates harsh conditions for yeast cells, leading to the formation of cell wall channels with the participation of glucan remodeling enzymes that remodel the β-glucans—the main constituents of the cell wall. The formation of such channels may be accompanied by the extrusion of mitochondrial material from the cell under stressful conditions.

## Methods

### Yeast and growth conditions

*Hansenula polymorpha* M14 and u23m25 strains were described previously (Agaphonov et al., 2005). The *H. polymorpha* cells were cultivated at 37 °C in the complex medium YEPD—1 % yeast extract, 2 % peptone, 2 % glucose, and 2 % agar when needed. The diploid strain of *H. polymorpha* was obtained by crossing of M14 and u23m25 strains.

### Treatment of yeast cells with amiodarone and their light microscopy

*Hansenula polymorpha* cells were grown in liquid YEPD medium at 37 °C overnight to the logarithmic phase. Amiodarone was diluted in DMSO, final concentration 0.4 %. Half-lethal concentration of amiodarone of 80 μM was chosen for the experiments. At this concentration, from 20 to 60 % of the *H. polymorpha* cells survive after 1 h of incubation, amiodarone at 20 μM does not affect, and 40 μM slightly reduces *H. polymorpha* cells survival (90–100 % survival). Amiodarone concentration of 120 μM causes death of 80 % or more of the *H. polymorpha* cells.

Cells were analyzed after 15 min of incubation with amiodarone by fluorescence microscopy and by electron-microscopic analysis, or after 60 min for gene transfer experiments. The longer time in the latter case was chosen to allow the completion of the transfer. For transmission electron microscopy, the cells were grown on 2 % YEPD agar plates overnight and then after amiodarone treatment were washed and fixed. All incubations were performed at room temperature.

The cells were stained either with propidium iodide (PI, Sigma Chemical) or with the DNA-specific fluorochrome 4′, 6′-diamidino-2-phenylindole (DAPI, Sigma Chemical). For PI staining of cultures, cells were pelleted by centrifugation for 5 min at 400*g*, the supernatant was discarded, and the cells were resuspended in PI solution (25 μg/ml in PBS) for 20 min at room temperature (Green et al. 1999). DAPI staining was performed for 10 min, the concentration of the dye being 300 μM (Morishita and Engebrecht 2005). The cells were grown to logarithmic phase on YPD medium, transferred into water, stained with 1 mM Mitotracker Red for 5 min, washed twice with water, and treated with either 0.4 % DMSO (control), or 80 μM amiodarone. Cells stained with MitoTracker Red and PI and were visualized with an upright Olympus BX51 microscope and U-MNG2 filter set (excitation 530–570 nm, 570-nm beam splitter filter, emission >590 nm). Cells stained with DAPI were visualized with a U-MNU2 filter set (excitation 360–370 nm, 400-nm beam splitter filter, emission >420 nm).

### Transmission electron microscopy

To facilitate cell-to-cell contacts and to secure the formed channels, colonies were treated with 80 μM amiodarone in DMSO to a final concentration 0.4 % directly on the agar plates for 15 min. After that, the amiodarone was removed by washing the agar plates, and the colonies were covered by liquid agar at the ambient temperature. Then the agar was cut into blocks. Small and thin agar blocks containing colonies treated (experimental samples) or untreated with amiodarone and treated with DMSO instead, final concentration 0.4 % (control sample), were washed twice in 0.1 M cacodylate buffer (pH 7.2). After embedding sections in the resin, we examined the cells comprising the colony. Fixation was performed in two steps: prefixation twice for 1 h in 5 % glutaraldehyde (with adding of 5 % DMSO v/v and 1.0 M sucrose) and post-fixation in 1.0 % OsO_4_ in cacodylate buffer pH 7.2 overnight at 4 °C. The blocks were dehydrated in a series of alcohol solutions with increasing concentrations (30, 50, 70, 80, 96, and 100 %), and then the blocks were embedded in araldite (Fluka). Ultrathin sections were prepared using an LKB 4800 ultramicrotome (LKB-Produkter, Sweden), contrasted with lead citrate in accordance with Reynolds (1963), and analyzed (Carl Zeiss electron microscope LEO912 AB, Germany, accelerating voltage 100  kV).

### Gene transfer experiments

Two milliliters of m14 (*leu2ade2URA3*) and u23-25 (*leu2ura3ADE2*) cells [OD600 = 0.2] were mixed and 80 μM amiodarone in DMSO was added to the mixture. After 5 min, the cells were centrifuged to facilitate cell-to-cell contacts and then incubated for 1 h. The cells were then resuspended and transferred onto YNB plates lacking adenine and uracil. The control cells were treated in the same way but with addition of DMSO instead of amiodarone.

### Flow cytometry

DNA content was determined according to (Epstein and Cross 1992) with one modification: treatment with RNAse was 20 min only because longer incubations reduced the signal. Briefly, the cells were fixed in 70 % ethanol overnight at 4 °C, then digested with 1 mg/ml RNAse (in 50 mM Tris–HCl (pH 8) for 20 min and stained with 50 μg/ml propidium iodide (in 180 mM Tris–HCl (pH 7.5), 190 mM NaCl, 70 mM MgCl_2_, overnight at 4 °C). Prior to measurements, the cells were ultrasonicated. Fluorescence and forward-angle light scattering were measured using a Becton–Dickinson FACScan instrument. In each experiment 10,000 cells were examined.

## Abbreviations

CW: cell wall
DAPI: 6′-diamidino-2-phenylindole
DIC: differential interference contrast
EM: electron microscopy
G: glucan layer
MP: mannoprotein layer
PI: propidium iodide
YNB: yeast nitrogen base
YEPD: yeast extract, pepton, d-glucose

## Footnotes

1 *Hansenula* was described to include different species that are included in the teleomorph genus *Ogataea* (Yamada et al. 1994). Because M14 and u23m25 strains ascend to *H. polymorpha* CBS 4732 strain (Agaphonov et al. 2005), they should be named *O. polymorpha* (sensu stricto) (Suh and Zhou 2010), but *H. polymorpha* is a more common name and is used in this report.
